# Genome-wide association analysis and transgenic characterization for amylose content regulating gene in tuber of *Dioscorea zingiberensis*

**DOI:** 10.1186/s12870-024-05122-4

**Published:** 2024-06-10

**Authors:** Shixian Sun, Binbin Guan, Yue Xing, Xiang Li, Lanlan Liu, Yanmei Li, Lu Jia, Shili Ye, Komivi Dossa, Li Zheng, Yunpeng Luan

**Affiliations:** 1 Yunnan Key Laboratory of Plateau Wetland Conservation, Restoration and Ecological Services, Southwest Forestry University, Kunming, 650224 China; 2 College of Landscape Architecture and Horticulture Sciences, Southwest Forestry University, Kunming, 650224 China; 3 Department of Life Science, Southwest Forestry University, Kunming, 650224 China; 4 The First Affiliated Hospital of Yunnan University of Traditional Chinese Medicine, Kunming, 650021 China; 5 Key Laboratory for Forest Resources Conservation and Utilization in the Southwest Mountains of China, Ministry of Education, Southwest Forestry University, Kunming, 650224 China; 6 Department of Life Technology Teaching and Research, School of Life Science, Southwest Forestry University, Kunming, 650224 China; 7 Faculty of Mathematics and Physics, Southwest Forestry University, Kunming, 650224 China; 8 UMR AGAP Institut, Univ Montpellier, CIRAD, INRAE, Institut Agro, Montpellier, 34398 France; 9 Eco-development Academy, Southwest Forestry University, Kunming, 650224 China; 10 Engineering Research Center for inheritance and innovation of Traditional Chinese Medicine, Kunming, 650034 China

**Keywords:** *S*tarch biosynthesis, Genome wide association study, Food quality, Yams, Food security

## Abstract

**Background:**

Amylose, a prebiotic found in yams is known to be beneficial for the gut microflora and is particularly advantageous for diabetic patients’ diet. However, the genetic machinery underlying amylose production remains elusive. A comprehensive characterization of the genetic basis of amylose content in yam tubers is a prerequisite for accelerating the genetic engineering of yams with respect to amylose content variation.

**Results:**

To uncover the genetic variants underlying variation in amylose content, we evaluated amylose content in freshly harvested tubers from 150 accessions of *Dioscorea zingibensis*. With 30,000 high-quality single nucleotide polymorphisms (SNP), we performed a genome-wide association analysis (GWAS). The population structure analysis classified the *D. zingiberensis* accessions into three groups. A total of 115 significant loci were detected on four chromosomes. Of these, 112 significant SNPs (log10(p) = 5, q-value **<** 0.004) were clustered in a narrow window on the chromosome 6 (chr6). The peak SNP at the position 75,609,202 on chr6 could explain 63.15% of amylose variation in the population and fell into the first exon of the ADP-glucose pyrophosphorylase (*AGPase*) small subunit gene, causing a non-synonymous modification of the resulting protein sequence. Allele segregation analysis showed that accessions with the rare G allele had a higher amylose content than those harboring the common A allele. However, *AGPase*, a key enzyme precursor of amylose biosynthesis, was not expressed differentially between accessions with A and G alleles. Overexpression of the two variants of *AGPase* in *Arabidopsis thaliana* resulted in a significantly higher amylose content in lines transformed with the *AGPase*-G allele.

**Conclusions:**

Overall, this study showed that a major genetic variant in *AGPase* probably enhances the enzyme activity leading to high amylose content in *D. zingiberensis* tuber. The results provide valuable insights for the development of amylose-enriched genotypes.

**Supplementary Information:**

The online version contains supplementary material available at 10.1186/s12870-024-05122-4.

## Background

Dioscorea species encompass over 600 species and represent one of the key tuber crops regarding their economic, pharmaceutical, and sociocultural importance [1]. Yam is positioned as the fourth most cultivated tuber crop behind potato, cassava, and sweet potato [2]. It serves as a fundamental dietary constituent and a significant economic revenue generator for an estimated 300 million people worldwide, mostly in tropical and subtropical regions [3].

Yam species, like other dietary fiber plants, contain high-amylose (resistant starch type 2), a useful prebiotic, which has a positive effect on gut microbiome, and can help with weight loss and obesity prevention [4–7]. For instance, high-amylose varieties of rice [8] and wheat [9] have a much lower glycemic load, which could be beneficial for diabetics. Additionally, high-amylose starch has been associated with various health benefits, including improved gut health, weight management, diabetes control, and potential cholesterol reduction, as indicated by studies involving animal models [10, 11]. Therefore, the genetic regulation of amylose content in crops such as rice [12], wheat [13], potato [14] and sweet potato [15] has sparked significant attention in the scientific community. Besides, amylose is widely utilized in pharmaceutical, food product, textile, and paper industries [12, 16].

The amylose content properties of yams have been studied for industrial applications. A study by Freitas et al. [17] found that defatted yam starch from *D. alata* cultivars had a significantly higher amylose content of 36.2% compared to 24.2% for cassava starch. This difference in amylose content is reflected in the different thermal and rheological properties of the two starches. Oscillatory rheometry revealed an initial gelatinisation temperature of 71 °C for yam starch, significantly higher than the 62 °C observed for cassava starch [17]. Furthermore, the gelatinisation process of yam starch was shown to require a higher activation energy, indicating a more energetically demanding process. Interestingly, the higher amylose content in yam starch also confers a slower gelatinisation rate at elevated temperatures and facilitates the formation of stronger gel structures upon cooling and retrogradation compared to cassava starch gels [17].

Furthermore, a study of starch granules isolated from five different yam species (*D. cayenensis*, *D. polygonoides*, *D. alata*, *D. rotundata*, and *D. esculenta*) revealed different characteristics in terms of amylose content, granule size, crystallinity and susceptibility to enzymatic digestion [18]. Amylose content varied significantly, with *D. cayenensis* having the highest amylose content at 26.5%. Notably, pronounced differences were observed in the digestibility of crude starches by porcine pancreatic α-amylase, with *D. esculenta* and *D. polygonoides* being the most susceptible to enzymatic degradation, while *D. cayenensis*, *D. rotundata* and *D. alata* starches exhibited greater resistance to digestion [18]. The amylose content of another yam species, *D. opposita* Thunb. cultivars, showed lower amylose content ranging from 20.74 to 25.94% compared to *D. alata* species [19].

Due to its unique properties, high-amylose starch is a valuable ingredient that offers a wide range of benefits in the food industry [20]. It has a higher melting temperature, limited granule swelling, lower water holding capacity, and a superior ability to form a gel during gelatinization [21]. Food products made from high-amylose starch, such as bread wheat and maize starch, can exhibit improved cooking quality [22, 23]. High-amylose starch possesses low enzymatic digestibility, which offers several nutritional and physiological benefits to humans such as improved glycemic control, increased dietary fiber intake, and reduced caloric value [20, 24]. High-amylose starch is also utilized in the encapsulation of probiotics and drugs, and in the formulation of oral rehydration treatments [25, 26].

As for low-amylose or amylose-free starches, they are used as thickening agents in food processing and papermaking industries [27]. In South East Asian countries such as Japan, South Korea and China, amylose-free cereals are widely consumed as part of the daily diet [28]. Low-amylose starches are also used to improve the shelf-life of products such as baked goods and snacks because they are less likely to become firm and grainy over time [29]. Low-amylose starches exhibit adhesive properties, making it a valuable alternative to petroleum-based adhesives production [30].

Despite these numerous advantages, the genetic architecture of amylose production in yam is not well understood. However, tremendous works have been done to unlock the genetic determinants of amylose biosynthesis using the plant model *Arabidopsis thaliana* [31], cereals including maize [32], wheat [13, 33, 34], barley [35], and rice [12, 16], and tubers such as sweet potato [15] and potato [14, 36].

**Fig. 1:**
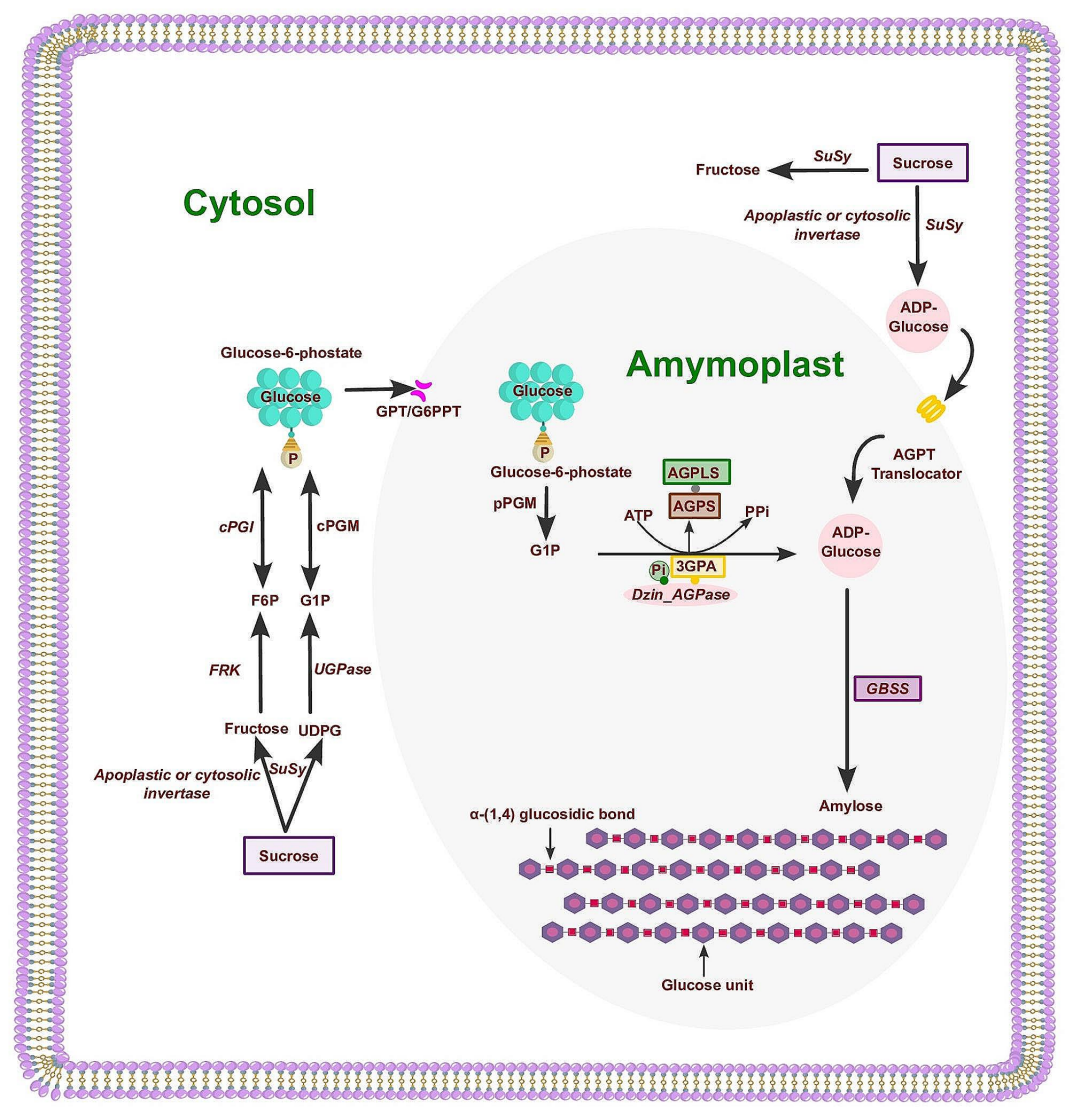
A simplified schematic view of the potential routes of amylose biosynthesis in *D. zingiberensis*. The diagram includes two compartments: the cytosol and the amymoplast. The stepwise reactions of sucrose synthase (*SuSy*), UGP-glucose pyrophosphorilase (*UGPase*) and plastidial phosphoglucomutase (*pPGM*) take place in the cytosol to convert sucrose to glucose-6-phosphate, which enters the amyloplast to be used for amylose biosynthesis. The enzymes involved in amylose biosynthesis in yam tubers include the precursor ADP-glucose pyrophosphorylase (*AGPase*) and granule-bound starch synthase (*GBSS*). Abbreviations are defined as follows Fructokinase (*FRK*); Glucose 1-phosphate (G1P); Glucose 6-phosphate/phosphate transporter (GPT/G6PPT); Fructose 6-phosphate (F6P); Cytosolic phosphoglucomutase (*cPGM*); cytosolic phosphoglucose isomerase (*cPGI*); inorganic phosphate (Pi); inorganic pyrophosphate (PPi); 3-phosphoglyceric acid (3-PGA); AGPase large subunit (*AGPLS*); AGPase small subunit (*AGPSS*).

Amylose biosynthesis in yam involves a series of enzyme-mediated steps and regulatory factors (Fig. 1). Sucrose from the phloem is cleaved into glucose and fructose, which are then converted to glucose-1-phosphate (G1P) [37]. G1P is activated by the enzyme ADP-glucose pyrophosphorylase (AGPase) to form ADP-glucose (ADPG), the immediate precursor of starch. AGPase catalyses this reaction, converting ATP to inorganic pyrophosphate (PPi) [38]. The catalytic activity of AGPase is inhibited by inorganic phosphate (Pi) and 3-phosphoglyceric acid (3-PGA) [39]. AGPase consists of two large (AGPLS) and two small (AGPSS) subunits, each with different functions. Granule-bound starch synthase (GBSS) elongates the glucan chains of amylose.

Genome wide association study (GWAS) has become very popular and is one of the main approaches to unlock the genetic basis of biological traits. In biomedical sector, GWAS helps scientists to identify genes associated with human diseases, enabling the development of suitable therapeutics [40]. In livestock, GWAS mainly aim to identify candidate genes related to important economic traits [41–43]. GWAS has also led to the discovery of large quantitative genetic loci associated with phenotypes of interest in several crops such as rice [44, 45], maize [46, 47], and peanut [48, 49].

In the past decade, the implementation of GWAS in yam breeding programs has emerged as a promising tool for accelerating genetic gains and enhancing the efficiency of breeding efforts. As for *D. alata*, GWAS has been employed to decipher the genetic architecture of tuber dry matter, oxidative browning [50], sex determination, cross-compatibility [51], flowering control [52], anthracnose, tuber size, tuber shape [53], and tuber flesh color [54]. Using an elite population of *D. rotundata*, loci associated with mosaic virus tolerance and yield tuber were identified using GWAS strategy [55]. While the greater yam, *D. alata*, has received considerable attention in genome-wide association studies (GWAS), interest has been growing recently in other species, including the bush yam (*D. praehensilis*) and white guinea yam (*D. rotundata*) [56, 57]. The first gene discovery efforts related to key traits in the bush yam, such as dry matter content, tuber flesh oxidation, and tuber flesh hardness have been performed [57]. As tuber quality is one of the key determinant of consumers preferences [58, 59], attention has been drawn to elucidating the genetic basis of yam food quality traits. Employing sensory quality evaluation and textural profile analysis, Asfaw et al. [56] identified putative genes underlying the textural properties of boiled and pounded yam food products derived from *D. rotundata*. Recently, taking advantage of whole genome sequencing of 127 genotypes of the greater yam, *D. alata*, Mota et al. discovered several genes involved some key tuber quality related pathways including starch and sucrose metabolism, pentose and glucuronate interconversions, and flavonoid biosynthesis [60]. Although the investigated traits are more likely related to tuber quality and agronomic performance, little is known about the genetic determinants of amylose content in yams.

Several genome-wide association studies (GWAS) have been conducted to understand the genetic regulation of starch quality traits, including starch content and pasting properties, in maize and barley [61, 62]. These studies have identified significant single nucleotide polymorphisms (SNPs) and candidate genes associated with starch traits, providing valuable insights into the genetic architecture of these traits. For example, in maize, GWAS has revealed the genetic control of starch content by multiple small effect quantitative trait loci (QTLs), and identified candidate genes related to starch pasting properties [61]. Similarly, in barley, GWAS has been used to identify novel putative alleles associated with total starch, amylose, and amylopectin content in grain [62].

*D. zingiberensis* is a dioecious perennial plant indigenous to southern China [63]. It has been discovered to contain over 70 bioactive compounds, exhibiting a diverse range of biological activities [64]. These include, but are not limited to, cardiovascular protection, anti-inflammatory responses, and anti-cancer properties [64, 65]. In the present study, we focused on the detection of genetic variants associated with amylose content, a key bioactive component with a wide range of applications. Molecular breeding techniques alongside CRISPR/Cas9-mediated gene knockout have been utilized to modulate the amylose content in major crops such as wheat [13] and rice [16]. Therefore, knowing the genomic regions and candidate genes underlying the biosynthesis of amylose in *D. zingiberensis* could pave the way for amylose-oriented genetic engineering.

## Results

### Analysis of amylose content variation in D. Zingiberensis panel

**Fig. 2:**
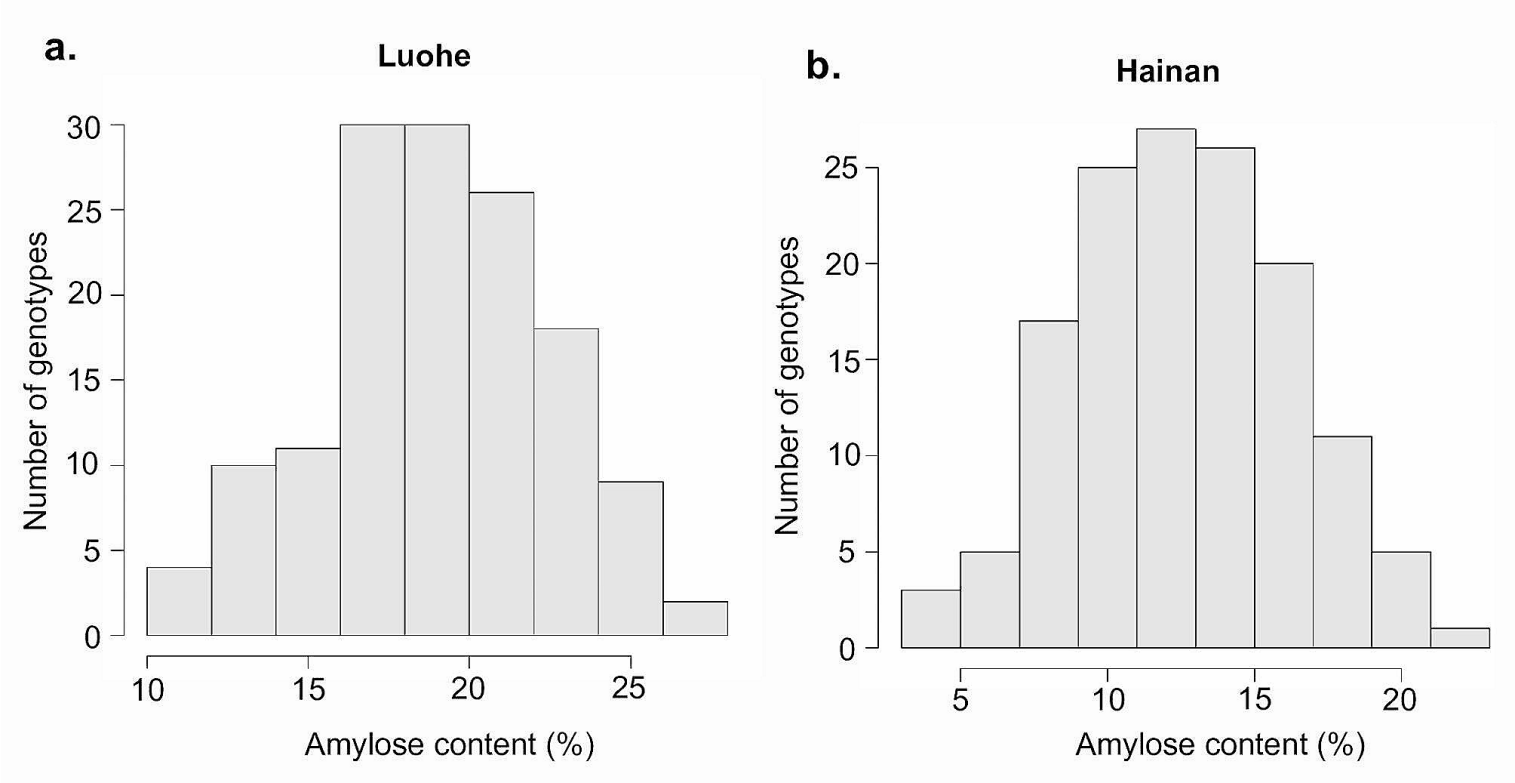
Distribution of amylose content data of *D. zingiberensis* accessions at Luohe **(a)** and Hainan **(b)**

A panel of 150 *D. zingiberensis* accessions was screened for their amylose content. The results indicate an approximatively normal distribution of the amylose content at both Luohe (*p*-value = 0.125) (Fig. 2a) and Hainan (*p*-value = 0.181) (Fig. 2b) environments in China.

The amylose content ranged from 9.11 to 32.25%, and from 10.23 to 30.78% at Luohe and Hainan, respectively (Table 1). The average amylose content among the accessions was significantly (*p*-value ≤ 0.001) higher in Luohe (20.41%) compared to Hainan (17.33%).

**Table 1:** Variability of the amylose content per location and contribution genotype, environment and genotype by environment effects

	Mean (%)	Range (%)	CV (%)	H^2^	G	E	G x E
Luohe	20.41	9.11–32.25	45.82	0.74	0.000381	0.00822	0.000517
Hainan	17.33	10.23–30.78	51.61				

G = Genotype, E = Environment, G x E : interaction, H^2^ = Broad-sense heritability

The analysis of variance also showed a highly significant (*p*-value ≤ 0.001) effect of environment, and genotype by environment factors on the amylose content. Besides, the broad-sense heritability was 74%, suggesting that variation in amylose content in *D. zingiberensis* tuber is substantially attributable to genetic causes.

### Population structure and principal component analysis

**Fig. 3:**
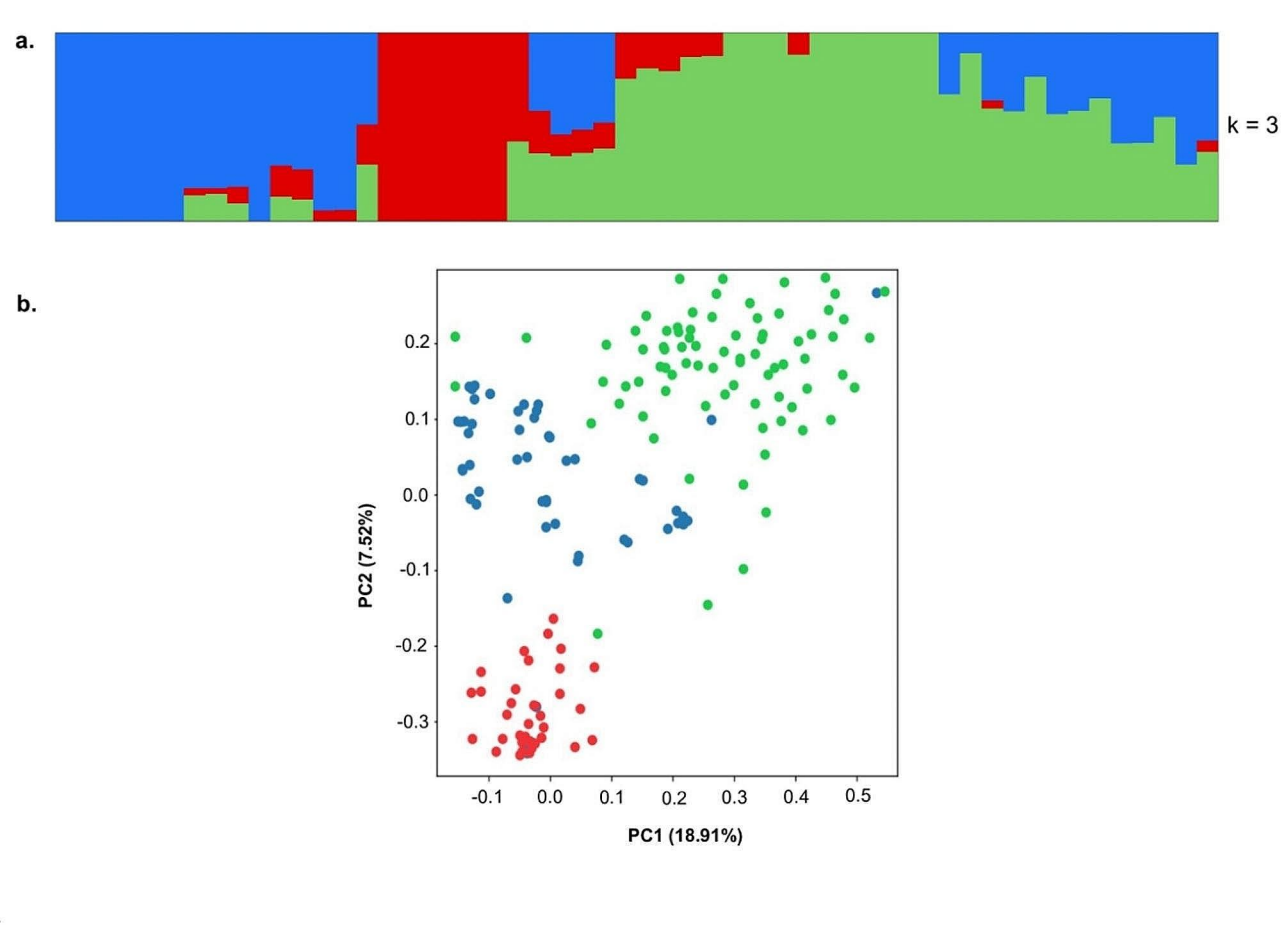
The results of the population structure **(a)** and principal component **(b)** analyses of the 150 *D. zingiberensis* accessions

Prior to conducting the genome-wide association analysis, we assessed the presence of putative sub-populations based on 30,000 high-quality SNPs. Population structure analysis revealed that the studied population can be divided into three sub-groups, with the majority of accessions being admixed (Fig. 3a; Supplementary Fig. 1). Principal component analysis (PCA) showed that the first two PC (PC1 and PC2) explained a total of 26.43% of the genetic variation in the population, with PC1 and PC2 explaining 18.91% and 7.52%, respectively. PC3 explained 4,61% of the genetic variation in the population (Supplementary Fig. 2). The low proportion of explained genetic variation suggests that the population of *D. zingiberensis* used in this study has low levels of population differentiation or substructure. Both STRUCTURE analysis (Fig. 3a), and PCA (Fig. 3b) confirmed the grouping of accessions into three sub-groups. Overall, the population exhibited a moderately structured pattern.

### Genome wide association study and identification loci controlling amylose variation

**Fig. 4:**
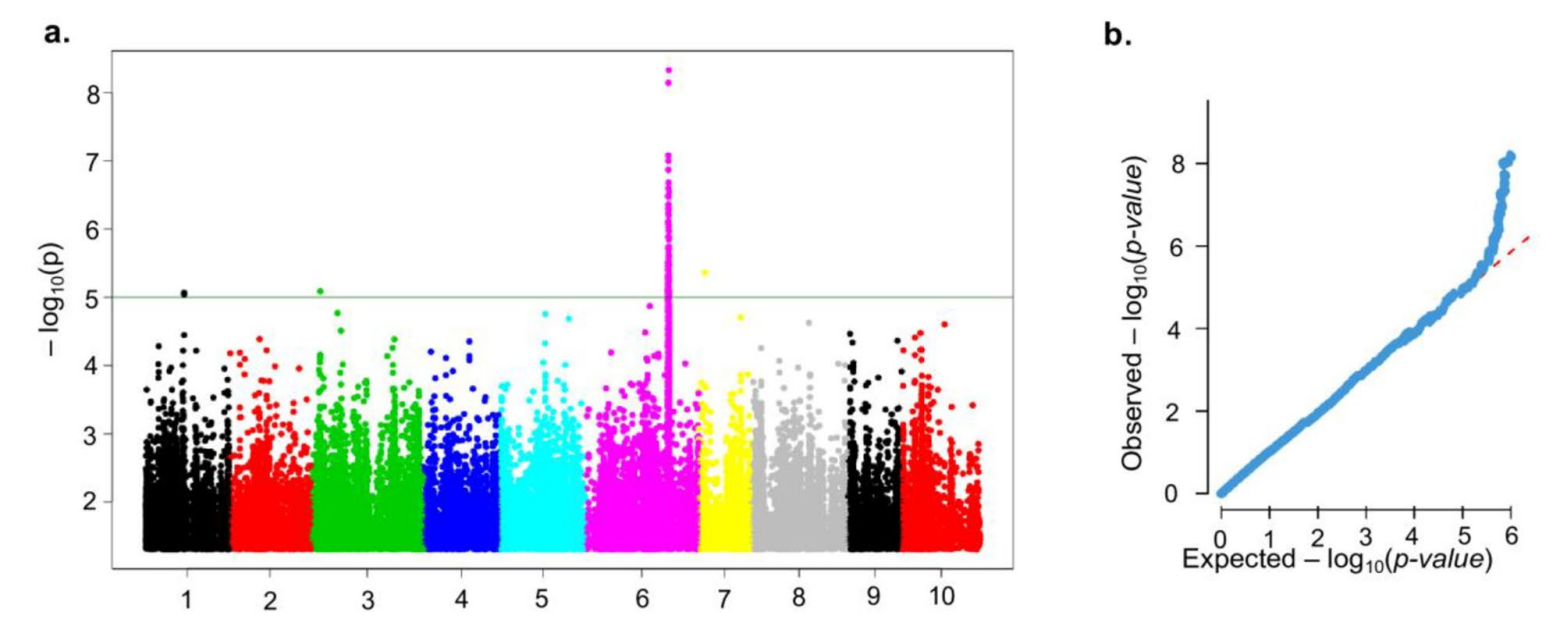
Genome-wide association mapping for amylose content in *D. zingiberensis*. Manhattan plot for amylose content **(a)**. Quantile-quantile plot for amylose content **(b)**

To identify the genetic loci accountable for the amylose content variation in *D. zingiberensis*, we performed a GWAS using a panel of 150 accessions. The resulting Manhattan plot highlighted at least four genomic regions containing 115 significant SNPs on the chromosomes chr1 (1), chr3 (1), chr6 (112), and chr7 (1) (Fig. 4a, Supplementary Table 1). The results of the quantile-quantile plot analysis (Fig. 4b) showed that the observed distribution did not deviate from the expected values to some extent. This result indicates a relative reduction of false positive (deviation from the expected values of the SNP markers) by the GWAS model.

The highest peak was observed on chr6 at the position 75609202 and could explain 63.15% of amylose variation in the population (Table 2). This result suggests that the variant Chr6_75609202 is a major locus controlling amylose content in *D*. *zingiberensis*. Other minor SNPs detected on chr1, chr3 and chr6 had very weak contributions to amylose content variation and did not fall into genic regions. Therefore, we focused our efforts on characterizing Chr6_75609202.

**Table 2:** Detected loci significantly associated with amylose content in *Dioscorea zingiberensis*

SNP	Chromosome	Position	Allele	*p*-value (-log10)	PVE^1^ (%)	Candidate gene
Chr6_75609202	6	75,609,202	A/G	8.23	63.15	*Dzin_AGPase*
Chr1_28410255	1	28,410,255	T/G	5.04	5.12	
Chr3_661488	3	661,488	C/A	5.12	4.09	
Chr7_8345231	7	8,345,231	A/G	5.21	5.24	

^1^ Phenotypic variation explained

**Fig. 5:**
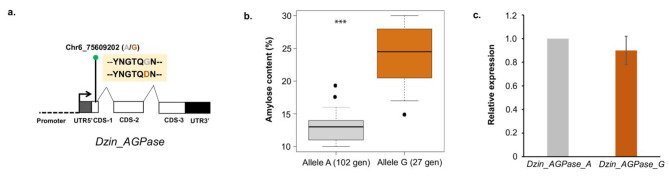
Characterization of *D*. *zingiberensis* AGPase (*Dzin_AGPase*) gene structure showing the location of the SNP Chr6_75609202 in the first exon. A single nucleotide polymorphism (SNP) was detected within this exon, leading to a non-synonymous alteration in the resulting protein sequence **(a)**. Comparative amylose quantification for accessions exhibiting A and G alleles **(b)**. Relative expression of the both versions of the gene via qRT-PCR experiment **(c)**

The SNP Chr6_75609202 was exactly located in the ADP-glucose pyrophosphorylase (AGPase) small subunit gene which is known to play a critical role in the starch biosynthesis [66, 67]. In-depth analysis the *D*. *zingiberensis* AGPase (*Dzin_AGPase*) gene revealed that the SNP is a non-synonymous (A/G) variant affecting the resulting protein sequence from glycine to aspartic acid (Fig. 5a). Moreover, the SNP Chr6_75609202 is located in the first exon of the gene (Fig. 5a).

Through mining the genotypic data, a total of 102 accessions presented the allele A while 27 harbored the allele G (Fig. 5b), indicating that A is the common allele. A comparative analysis of the amylose content in both groups showed a highly significant (p-value < 0.001) difference in the G allele group compared to the A allele group. Thus, the G allele could be considered the favorable allele for higher amount of amylose production in *D*. *zingiberensis* tuber.

Since the genetic variation fell into a genic region, we tested whether it impacts on the gene expression level. We performed a qRT-PCR experiment with five accessions harboring the G allele and five accessions with the A allele. The results (Fig. 5c) revealed a similar relative expression level of *Dzin_AGPase* for both alleles, indicating that the discovered variant did not impact the gene transcription.

### Functional analysis of the Dzin_AGPase gene

**Fig. 6:**
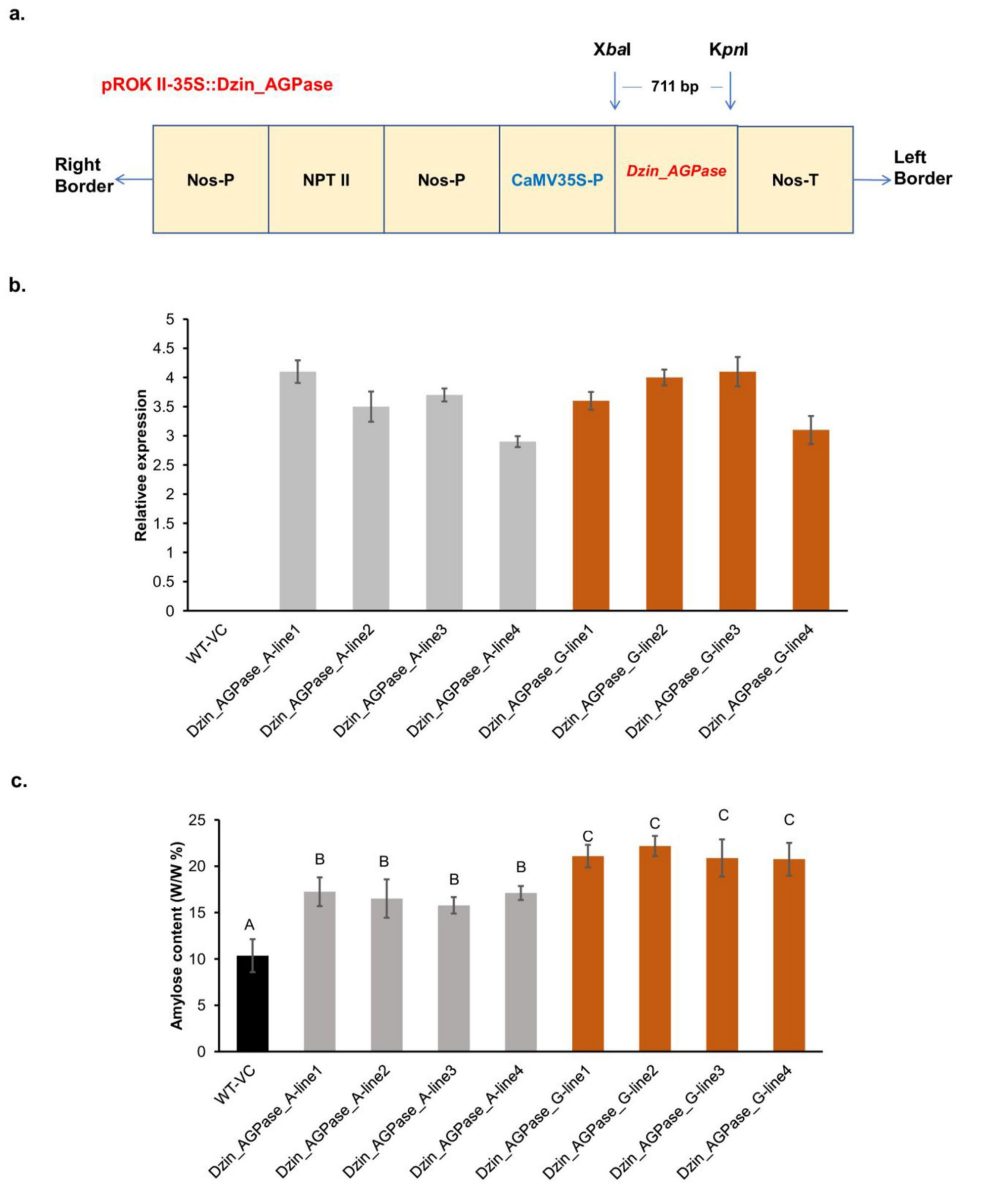
Generation of *Arabidopsis thaliana* transgenic plants for the overexpression of the *DZin_AGPase* gene **(a)**. The construct of plasmid contains the *Dzin_AGPase* gene, the CaMV35S promoter, and NOS terminator. The NPT II was employed as a selective marker. RB, right border; LB, left border; NOS-P, nopaline synthase promoter; NOS-T, nopaline synthase terminator. Relative expression **(b)** and amylose content quantification **(c)** from the T3 generation transgenic plants. Four lines for each allele were selected. WT-VC is vector control (transformed with empty vector). Mean comparison significance of the amylose content was depicted with the letter A, B, and C

To validate the predicted function of the candidate *Dzin_AGPase* gene, an *Arabidopsis thaliana*-based transformation was conducted. The full-length cDNA of the two variants of *Dzin_AGPase* were ligated into different pROK II-35 S vector promoted by the CAMV35S promoter (Fig. 6a). From the positive T3 overexpressing plants, leaves from four lines of each allele type with seven replicates each, were harvested for amylose and gene expression profile assessment.

The relative expression profile (Fig. 6b) of the transgenic lines indicates a highly similar expression level among the lines for both alleles of the *Dzin_AGPase* gene. However, the amylose content (Fig. 6c) was significantly higher in the G allele that in the A allele overexpressing lines. From the relative expression and amylose quantification results, we deduced that the variant did not impact on the transcription and function of the protein but seemingly the enzyme activity is increased when it comes to the G allele, resulting in higher amylose content.

## Discussion

Amylose is a valuable resource with many applications covering food, paper, textiles, adhesives, and health care industries [68, 69]. The genetic basis of amylose content, a component of starch, has been investigated in tuberous crops, such as potato [70], sweet potato [15], cassava [71] and non-tuber crops, including maize [32], wheat [33], and rice [16]. The improvement of amylose content in tubers for both consumption and industrial purposes has been a major objective for breeders. Taking advantage of a large panel combined with a high-quality genotyping dataset, we were able to dive into the genetic variants modulating amylose biosynthesis in *D. zingiberensis* tuber using a genome-wide association study (GWAS) approach.

In the present study, not only the genotype effect but also the genotype-by-environment effect were highlighted, influencing the amylose content in the studied *D. zingiberensis* panel. This implies that the environmental component is also a key determinant of amylose variation and should be considered when developing stable and high-yield amylose content genotypes. Similarly, a significant environmental effect was also found for greater yam (*Dioscorea alata*) on tuber quality traits including flesh colour, tuber dry matter, oxidative browning, skin texture and shape regularity [50, 72], highlighting the importance of considering environmental effect for future research and breeding programs.

From GWAS, we identified four putative loci associated with amylose content variation in *D. zingiberensis*. Among these, three minor SNPs had very weak contribution and were not linked to any candidate gene. Interestingly, one SNP was located in the first exon of the ADP-glucose pyrophosphorylase gene. The AGPase is well-known to be one of the key precursors at the upstream step of starch production [37]. Its role is to catalyze the conversion of 1-P-glucose into ADP-glucose to enable the biosynthesis of both amylose and amylopectin [66, 67, 73]. The regulatory properties of the ADPase gene in amylose production was firstly demonstrated in potato through overexpression approach [74]. This approach has gained popularity within some major cereals crops, with a specific focus on maize [75, 76], wheat [77, 78], and rice [79, 80]. Overexpression of AGPase in maize resulted in an increase in starch (amylose and amylopectin) production and seed weight [75]. An increase in seed yield was also observed in overexpressed AGPase wheat [77] and rice [79, 80] lines. In addition to the regulatory role of AGPase with regard to amylose production, it has also been shown to have an impact on photosynthesis and carbon metabolism. By increasing the availability of ADPglucose for starch biosynthesis, AGPase enhances the capacity of the leaf to store carbon, which in turn, stimulates photosynthesis by allowing more efficient use of light energy [78].

We also uncovered that allelic variation in the *DZin_AGPase* is associated with amylose content level in the studied *D. zingiberensis* panel. Genotypes with the G allele exhibited approximately two-fold higher amylose content than the A version of the gene. Surprisingly, the expression levels of both alleles are in the same range, indicating that both versions of the genes are equitably expressed but somehow, the amylose content in genotypes with G allele is higher. The presence of non-synonymous changes in the protein sequence between the A and G alleles of *AGPase*, suggests that this change may lead to functional differences. The absence of evidence for differential expression of the two alleles in transcriptome data may indicate that post-transcriptional or post-translational modifications may be responsible for observed differences in amylose content. Further investigation is required to elucidate the mechanisms underlying these differences and their potential implications for breeding programs aimed at developing cultivars with higher amylose content.

The allelic variation associated with the amylose content in the panel and transgenic lines comforts the rate-limiting enzyme function of the AGPase. In fact, the AGPase subunits interaction [81–84], and specifically, amino acid motifs [85, 86] responsible for allosteric regulation are thought to confer to AGPase, the capabilities of starch level regulation. Therefore, AGPase has become a prime target for enzyme engineering, to increase starch content in some food crops including common wheat [87], maize [75], rice [79], and potato [86].

To functionally test the role of the *DZin_AGPase*, we proceeded to a transgenic experiment using *Arabidopsis thaliana* as plant model. *DZin_AGPase* overexpression showed a relatively high proportion of amylose content for the G allele compared the A allele. Therefore, the G version will likely be valuable to boost the production of amylose in *D. zingiberensis*. In-depth genetic improvement of amylose content through CRISPR-Cas9 for example, might be a promising avenue to explore. Meanwhile, for the tetraploid potato, amylose-free genotype has been recently developed by CRISPR/Cas9-mediated mutagenesis [88]. Besides, the control of the amylose content in sweet potato has also been successfully conducted [15]. It is worth noting that these two success stories exclusively relied on the granule-bound starch synthase gene editing, which is the amylose encoding enzyme. Noticeably, the granule-bound starch synthase gene has not been found in the present study. Knowing that the accumulation of amylose and the expression of key enzymes follows a diurnal pattern [89, 90], timeline transcriptome profiling of the tuber following expansion of the tuber might potentially enlighten others key enzyme master players.

## Methods

### Plant materials and field experiments

To identify the genomic regions associated with amylose content in *D. zingiberensis*, a panel of 150 accessions was utilized in a field experiment conducted in two locations in China: Hainan (18° 56’ 22’’ North and 109° 29’ 3’’ East) and Luohe (33° 34’ 18’’ North and 114° 2’ 7’’ East). The experiment was designed as a randomized complete block with three replicates. Within each block, five replicates of each accession were sown on ridges, and all recommended in-field cultural practices were applied at both sites until tuber harvest, which occurred upon leaf senescence. The plant materials were formally identified by Prof Yunpeng Luan and all germplasms are conserved as vitro-plant at the Genebank of Southwest Forestry University. No permission is required to work on this species. The accessions originated from Southern China within relatively similar agroecological zones.

### Starch isolation

Freshly harvested yam tubers were processed for starch isolation following a modified protocol of Farhat et al. [91]. Briefly, the rhizomes were washed, peeled, and cut into small pieces. A total of 100 g of the slices were ground with 900 mL of 1% sodium chloride solution in a commercial blender (Waring Commercial, Stamford, Connecticut, USA) for two minutes. The resulting slurry was then passed through a 106 μm pore diameter sieve (Fisher Scientific, Waltham, Massachusetts, USA) to remove any solid particles. The obtained suspension was left to settle overnight (12 h) at room temperature to allow for starch precipitation. The supernatant was decanted, and the starch pellet was centrifuged at 3000 g for 10 min. The top brown layer was carefully removed, and the starch was subsequently resuspended in solutions of 1% w/v sodium chloride and de-ionized water, respectively. For each washing step, the solution was re-centrifuged two to three times. Finally, the freshly isolated starch was then dried at 60 °C in an oven (Precision Scientific, GCA Equipment Corporation, Madison, Wisconsin, USA), ground, and stored at room temperature in a glass container prior to usage.

### Amylose Content quantification

The amylose content was quantified according to the iodine binding colorimetric methodology outlined in Jiang et al. [92] study with modifications. Firstly, a quantity of 10 mg of the previously prepared starch was mixed with 2 mL of Dimethyl sulfoxide and subjected to heating at 85 °C for 15 min. The dissolved starch was then diluted with deionized water to attain a final volume of 25 mL. A volume of 1 mL of the starch solution was transferred into a 50 mL flask followed by the addition of 5 mL of iodine. Lastly, the optical absorbance was recorded at 620 nm using a Cary 60 UV-Vis spectrophotometer (Agilent, Santa Clara, California, USA). Triplicate apparent amylose content was carried out for each accession.

### Amylose Content Data Evaluation and Statistical Analysis

The collected data were checked for normality through frequency distribution histogram plot, and Shapiro-Wilk test using R program v.4.2.2 [93]. The mean, range, and coefficient of variation were also computed in R program. Subsequently, an analysis of variance was performed following the model:

1 $$\begin{gathered}{y_{ijk}} = {\text{ }}\mu + {\text{ }}Lo{c_i} + {\text{ }}\operatorname{Re} {p_j}\left( {Lo{c_i}} \right) \hfill \\\,\,\,\,\,\,\,\,\,\,\,\,\,\,\,\,\, + {\text{ }}Ge{n_k} + {\text{ }}Lo{c_i} \times {\text{ }}Ge{n_k} + {\text{ }}{\varepsilon _{ijk}} \hfill \\ \end{gathered}$$

Where ${\text{y}}_{\text{ijk}}$ is the observed value of the amylose content in the i^th^ location, j^th^ block for the k^th^ genotype, $\mu$ is the overall general mean, ${\text{Loc}}_{\text{i}}$ is the effect of the i^th^ location, ${\varepsilon _{{\text{ijk}}}}$ is the experimental pooled error effect, ${\text{Rep}}_{\text{j}}\left({\text{Loc}}_{\text{i}}\right)$ is the effect of j^th^ block within i^th^ location,${\text{Gen}}_{\text{k}}$ is the effect of k^th^ genotype, ${\text{Lo}}{{\text{c}}_{\text{i}}} \times {\text{ Ge}}{{\text{n}}_{\text{k}}}$ is the effect of the interaction between the i^th^ location and the k^th^ genotype, and ${\varepsilon _{{\text{ijk}}}}$ is the experimental pooled error.

The variance components were computed by fitting the mixed linear model with genotype, location, and genotype by location factors as random effect using lme4 package [94]. Furthermore, the heritability (${H}^{2}$) was calculated as:

2 $${H}^{2}= \frac{{\sigma }_{g}^{2}}{{{\sigma }_{g}^{2} +{\sigma }_{ge}^{2}/nLoc+\sigma }_{\epsilon }^{2}/(nLoc \times nRep)}$$

where ${{\sigma }}_{\text{g}}^{2}$ is the genotype variance component, ${{\sigma }}_{\text{g}\text{e}}^{2}$ is the genotype by environment interaction variance component, nLoc is the number of environments and the nRep the number of replicates.

The best linear and unbiased predictors (BLUPs) values calculated from the model, served for the downstream GWAS analysis. Normality test was conducted with shapiro.test() in R program v.4.2.2.

### SNP Genotyping

From 100 g young leaves tissues, we extracted the genomic DNA for each accession with ImaSpin® Genomic DNA Kit (Imagene Bioscience, China) following the manufacturer’s protocol. The quality of the DNA was checked using a Nanodrop 8000 spectrophotometer (Thermo Fisher Scientific, Waltham, MA, USA). A volume of 30 mL of DNA was pipetted into 96-well PCR plates, and genotyping-by-sequencing (GBS) was conducted following a 96-plex Pst I GBS protocol [95]. Briefly, the DNA of each accession was digested with the restriction enzyme PstI (New England Biolabs, Beijing, China). Restriction cutting sites were ligated with adapters (barcodes) with the T4 ligase. The ligated products were then pooled together. Single-end sequencing was performed using an Illumina HiSeq2500 instrument (Illumina Inc. San Diego, CA, USA).

The generated raw reads were processed (sorting, demultiplexing and trimming) using the TASSEL GBS v2 pipeline [96] (Supplementary Table 2). The mapping onto the reference genome [97] was performed using the Burrows–Wheeler alignment (BWA) v0.7.17 (Li and Durbin, 2009), and the SNPs were called with DiscoverySNPCallerPluginV2 of the TASSEL GBS v2 pipeline yielding 2.3 M SNPs. A minimum locus coverage (mnLov) was set to 0.1, while other parameters were maintained to default settings. Monomorphic sites, SNPs with missing data > 20%, and with minor allele frequency (MAF) < 0.01, were excluded using vcftools v0.1.16 [98]. The resulting data was imputed with Beagle v4.1 [99] yielding (842,000 SNPs), and a second round of SNPs with MAF < 0.01 were filtered out prior to the downstream analyses. Out of 54,000 SNPs, 30,000 high-quality SNPs were retained for downstream analyses.

### Population structure analysis

The population genetic structure of the 150 accessions was inferred by using a Bayesian model-based method embedded in STRUCTURE v2.3.4 [100]. The number of population clusters was predetermined as k ranging from 1 to 10. We applied five independent runs for each k. Each run involved a total of 100,000 Markov chain Monte Carlo iterations after a burn-in period of 100,000 iterations. We determined the best k population following the Evanno ΔK method. Besides, the principal component analysis (PCA) was performed using the Genomic Association and Prediction Integrated Tool (GAPIT) [101] in the R program v.4.2.2 [93].

### Genome-wide Association analyses

To find out putative candidate genomic regions related to the amylose content accumulation in tuber, the association phenotype-genotype analysis was performed using the GAPIT package following the mixed linear model (MLM) option [102]. Both kinship matrix and PCA matrix were employed as random and fixed effects, respectively. Since we retained a set of 30,000 high-quality SNPs meeting the stringent filtering criteria, the genome wide significant threshold was set to 5 following the calculation -log_10_(p) with *p* = 1/30,000. The Manhattan and qq plots were rendered using the qqman package [103].

### Quantitative RealTime PCR (qRTPCR) analysis

To evaluate the expression of the candidate gene, a qRT-PCR experiment was conducted in an Applied Biosystems™ 7500 Real-Time PCR machine (Thermo Fisher Scientific, Waltham, Massachusetts, USA) with a SYBR Green PCR Master Mix (Tiangen Biotech, Beijing, China). Total RNA was extracted with RNAprep Pure Plant Kit (Tiangen Biotech, Beijing, China), and the RNA was transcribed with the help of a Quantscript Reverse Transcriptase Kit (Tiangen Biotech, Beijing, China). A primer pair (5’-AGAATCTAGACCACTTAC-3’; 5’-CTCTAGGTACAGTCTCA-3’) was designed, and the PCR experiment was conducted with the following conditions: denaturation step at 95 °C for 10 min, annealing step with 40 cycles at 95 °C for 15 s, and the extension step at 60 °C for 1 min. The relative expression of the candidate genes was quantified following the comparative C_T_ method [104]. Three replicates were applied for each gene variant, and the expression data were normalized against those of *D. zingiberensis* actin gene sequence (NCBI GenBank accession: JN693499).

### Arabidopsis transgenics experiment

To functionally characterize the candidate gene *Dzin_AGPase*, we extracted the protein coding region from two genotypes, each having different alleles. The construct design and cloning were performed using the pROK II-35 S vector. The Arabidopsis transformation was performed following the floral dip method as outlined by Clough and Bent [105] using *Agrobacterium tumefaciens* strain LBA4404. The overexpressing plants (T3 homozygous lines) were then transferred into larger pots and maintained in greenhouse. The gene expression and the amylose content quantification were executed in accordance with the aforementioned methods.

## Conclusions

In the present study, we report for the first time, a major locus associated with amylose content in a non-model plant *D. zingiberenisis*. *DZin_AGPase*, a starch rate-limiting enzyme, exhibited allelic variation with the G allele associated to higher amylose content. Overexpression of the two *DZin_AGPase* alleles using *Arabidopsis* transgenic plants corroborated the higher amylose content for the G allele. Our findings provide a valuable foundation for developing new varieties with desired amylose content levels. This study can also contribute to improving the nutritional quality of yam-based foods, as amylose content affects their digestibility and glycemic index. Future research can expand on our findings by examining the functional roles of the identified genetic variants using CRISPR-Cas9.

### Electronic supplementary material

Below is the link to the electronic supplementary material.

Supplementary Material 1

Supplementary Material 2

Supplementary Material 3

Supplementary Material 4

Supplementary Material 5

## Data Availability

The raw sequencing data are available at NCBI SRA under the project number: 716093 (https://www.ncbi.nlm.nih.gov/bioproject/716093). Codes and scripts used for data analysis are available in Supplementary file 1. Any intermediary file and all phenotypic data can be provided upon request to the corresponding author.
