# Supplementary material for: Genome-wide association analysis and transgenic characterization for amylose content regulating gene in tuber of Dioscorea zingiberensis
Source: BMC Plant Biol. 2024 Jun 10;24:524. doi: 10.1186/s12870-024-05122-4 (PMC11163818; doi:10.1186/s12870-024-05122-4)
Supplement: Supplementary file 1 — Supplementary Material 1 [file 12870_2024_5122_MOESM1_ESM.docx]

**Supplementary file 1:** Codes and scripts used for data analysis

**descriptive_statistics_normality_test**

# Load required libraries

library(stats) # for statistical tests

# Assuming you have your data loaded or imported into a dataframe named 'data'

data = read.csv("amylosedata.csv", h=T)

# Frequency distribution histogram plot

hist(data$amylose,

main = "Luohe",

xlab = "Amylose content (%)",

ylab = "Number of genotypes",

col = "grey",

border = "black")

# Shapiro-Wilk test for normality

shapiro.test(data$amylose)

# Compute mean, range, and coefficient of variation (CV)

mean_value <- mean(data$amylose)

range_value <- max(data$amylose) - min(data$amylose)

cv_value <- sd(data$amylose) / mean(data$amylose) * 100

# Print the results

cat("Mean:", mean_value, "\n")

cat("Range:", range_value, "\n")

cat("Coefficient of Variation (CV):", cv_value, "%\n")

GWAS GAPIT in R

###########################################

#Download and install GAPIT

source("http://zzlab.net/GAPIT/GAPIT.library.R")

source("http://zzlab.net/GAPIT/gapit_functions.txt")

#set the working directory

setwd("D:/analysis/GAPIT/")

#GWAS with three models for comparison

myGAPIT <- GAPIT(

Y=myY,

G=myG,

PCA.total=3,

model=c( "MLM", "FarmCPU", "Blink"),

Multiple_analysis=TRUE)

# GWAS only MLM method

myGAPIT_MLM <- GAPIT(

Y=myY,

G=myG,

PCA.total=3,

model="MLM")

**SNP genotyping in Tassel**

#install tassel5

wget https://bitbucket.org/tasseladmin/tassel-5-standalone/get/V5.2.72.zip

unzip V5.2.72.zip

#create four folders named: fastq, key, output, referenceGenome, using the command below:

mkdir fastq key output referenceGenome

# run tassel

cd /home/analysis/tassel5

# run the GBSSeqToTagDBPlugin :

./run_pipeline.pl -Xms100G -Xmx180G -GBSSeqToTagDBPlugin -e ApeKI -i /home/analysis/fastq/ -db /home/analysis/output/GBSv2.db -k /home/analysis/key/Pipeline_Testing_key.txt -kmerLength 64 -mnQS 20 -c 5

# run the TagExportToFastqPlugin

./run_pipeline.pl -Xms100G -Xmx180G -TagExportToFastqPlugin -db /home/analysis/output/GBSv2.db -o /home/sam/DEFI/gbs_test/output/tagsForAlign.fa.gz

#create a folder INDEX

mkdir /home/analysis/INDEX

# Run BWA software to create an index base named 'IndexTest' from the reference genome 'd_alata_ref.fasta' to be put in the folder INDEX

bwa index d_alata_ref.fasta d_alata_ref

#After the indexes have been created, the alignment command can be run

bwa mem d_alata_ref input_reads_pair_1.fastq input_reads_pair_2.fastq -t 16 > bwa_mem_alignments.sam

#SAMToGBSdbPlugin

cd tassel5

./run_pipeline.pl -SAMToGBSdbPlugin -i /home/analysis/output/bwa_mem_alignments.sam -db /home/analysis/output/GBSv2.db -aProp 0.0 -aLen 0

#DiscoverySNPCallerPluginV2

cd tassel5

./run_pipeline.pl -DiscoverySNPCallerPluginV2 -db /home/analysis/output/GBSv2.db -mnLCov 0.1 -deleteOldData true

#ProductionSNPCallerPluginV2

cd tassel5

./run_pipeline.pl -ProductionSNPCallerPluginV2 -db /home/analysis/output/GBSv2.db -e ApeKI -i /home/analysis/fastq/ -k /home/analysis/gbs_test/key/Pipeline_Testing_key.txt -kmerLength 64 -o /home/analysis/output/alata.vcf

# check snp quality SNPQualityProfilerPlugin using all individuals:

./run_pipeline.pl -SNPQualityProfilerPlugin -db /home/analysis/output/GBSv2.db -statFile /home/analysis/output/SNPQualityStatsAll.txt

# generate a new VCF file containing the filtered SNPs according to the specified criteria

vcftools --vcf input_alata.vcf --max-missing 0.2 --maf 0.01 --min-alleles 2 --max-alleles 2 --recode --out output_alata_filtered

# imputation with Beagle v4.1

java -Xmx100G -jar beagle.jar \

gt=output_alata_filtered.vcf \

out=output_alata_filtered.imputed \

impute=true

# second round filtering

vcftools --vcf output_alata_filtered.imputed.vcf --maf 0.01 --recode --out output_alata_filtered.imputed.2nd.filtered
