# Supplementary material for: Genome-wide association analysis and transgenic characterization for amylose content regulating gene in tuber of Dioscorea zingiberensis
Source: BMC Plant Biol. 2024 Jun 10;24:524. doi: 10.1186/s12870-024-05122-4 (PMC11163818; doi:10.1186/s12870-024-05122-4)
Supplement: Supplementary file 2 — Supplementary Material 2 [file 12870_2024_5122_MOESM2_ESM.pptx]

## Slide 1
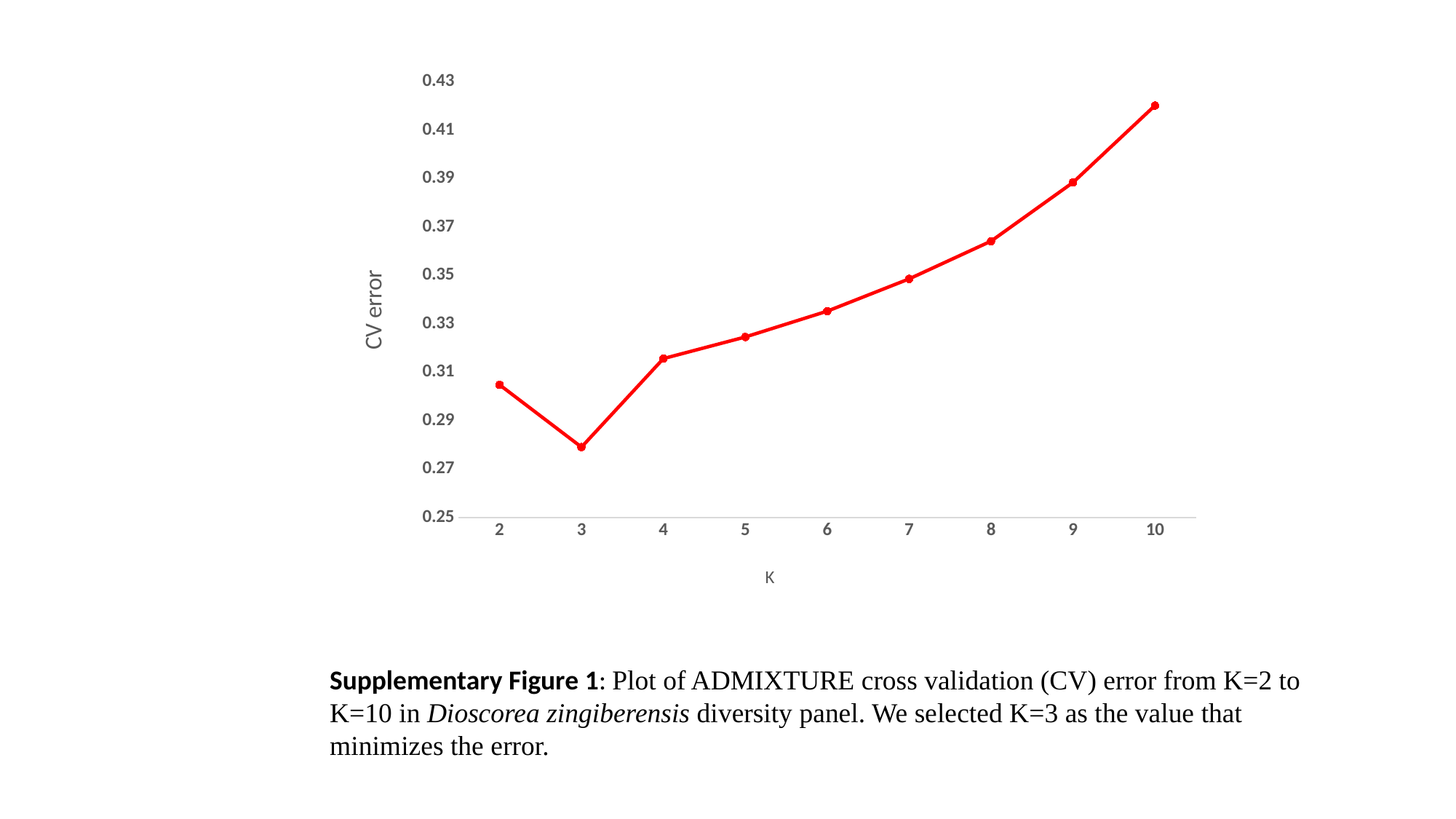

### Chart
| Category | |
|---|---|
| 2 | 0.30486 |
| 3 | 0.27911 |
| 4 | 0.31567 |
| 5 | 0.32462 |
| 6 | 0.33528 |
| 7 | 0.34862 |
| 8 | 0.36422 |
| 9 | 0.38847 |
| 10 | 0.42022 |Supplementary Figure 1: Plot of ADMIXTURE cross validation (CV) error from K=2 to K=10 in Dioscorea zingiberensis diversity panel. We selected K=3 as the value that minimizes the error.
