# Supplementary material for: Genome-wide association analysis and transgenic characterization for amylose content regulating gene in tuber of Dioscorea zingiberensis
Source: BMC Plant Biol. 2024 Jun 10;24:524. doi: 10.1186/s12870-024-05122-4 (PMC11163818; doi:10.1186/s12870-024-05122-4)

Supplementary Figure 2: Principal component analysis (PCA) of the 150 *D. zingiberensis* accessions. PC1 and PC3 are displayed.

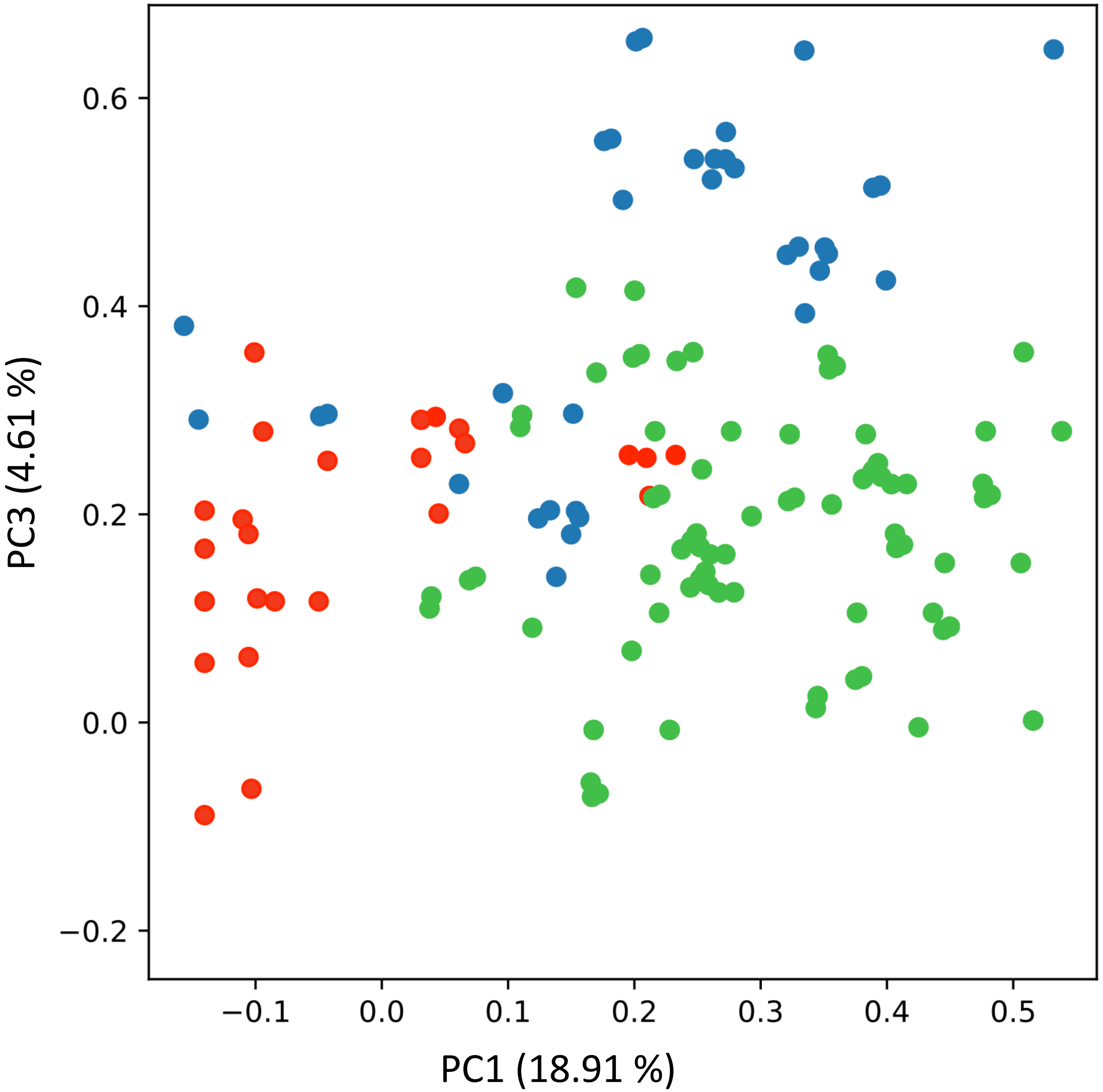

Supplement: Supplementary file 3 — Supplementary Material 3 [file 12870_2024_5122_MOESM3_ESM.pdf]
